# Drug-induced liver injury due to tofacitinib: a case report

**DOI:** 10.1186/s13256-023-03821-4

**Published:** 2023-03-18

**Authors:** Masoud Mardani, Jafar Mohammadshahi, Sara abolghasemi, Roghayeh Teimourpour

**Affiliations:** 1 Infectious Diseases and Tropical Medicine Research Center, Shahid Beheshti University of Medical Sciences, Tehran, Iran; 2 Department of Infectious Diseases, School of medicine, Ardabil University of Medical Science, Ardabil, Iran; 3 Department of Microbiology, School of medicine, Ardabil University of Medical Science, Ardabil, Iran

**Keywords:** Tofacitinib, Alopecia, Anorexia, Liver, Case report

## Abstract

**Background:**

Drug-induced liver injury is an acute or chronic liver damage in response to drugs, herbals, and any chemical compound.

**Case presentation:**

In the present work, liver failure following the use of tofacitinib was reported. The patient was an 18-year-old iranian woman without any history of underlying disease. She complained of alopecia areata, and tofacitinib was administered for disease management. Following adherence to tofacitinib medication, partial recovery was obtained. At the time of hospitalization, the patient had a stable condition and only anorexia, jaundice, and elevation of liver enzymes were reported. During hospitalization, liver injury progressed and liver transplantation was suggested. After drug-induced liver injury diagnosis, the use of the drug was discontinued and the patient underwent supportive treatment. The patient recovered without any severe sequelae.

**Conclusions:**

Tofacitinib is a Janus kinase inhibitor that is useful in the treatment of disorders such as rheumatoid arthritis, psoriatic arthritis, and ulcerative colitis. Until now, the severe side effect of this drug has not been reported and in most cases it is used as a last resort, but here we report a rare side effect of this drug.

## Background

The liver plays an essential role in concentrating and metabolizing drugs. Many synthetic drugs, herbals, toxins, and even food supplements can lead to liver damage. This damage can occur immediately or months or years after taking the drug. In most cases, there is an increase in liver enzymes in the blood, which can be accompanied by symptoms. Liver involvement can be acute, chronic, cholestatic, and even lead to liver transplantation. In general, 10% of acute hepatitis or jaundice cases are related to the toxic effects of drugs [1]. Drug-induced liver injury (DILI) can be the result of a direct effect of the drug itself or caused by toxic metabolites from the drug metabolization in the liver. Studies have shown that the rate of DILI has increased worldwide since 2010. The prevalence of DILI is varied in different parts of the world, but the highest incidence rate is in Asia (17.82 per 100,000) and the lowest is in America (1.72 per 100,000 people) [2]. Early diagnosis and critical management of DILI are essential in dealing with these cases to save liver function and eliminate the need for liver transplantation.

## Case presentation

An 18-year-old iranian woman with anorexia and yellow discoloration of the conjunctiva and urine presentation was admitted to the Taleghani hospital in Tehran. At the time of admission, the patient had a stable condition but she was icteric and complained of loss of appetite for the last 2 weeks. Preliminary lab tests showed a high rate of total bilirubin (16.4 mg/dl) and elevated liver transaminase enzymes with normal blood cells. Medical history examination revealed that she had received tofacitinib (5 mg/daily) for treatment of alopecia areata for 5 months. Any history of a particular disease, alcohol consumption, and other medications except tofacitinib was not reported. Three months after beginning tofacitinib medication, a partial recovery in the alopecia lesions was seen, and a series of tests, including serum glutamic-oxaloacetic transaminase (SGOT, 335 IU/ml), serum glutamic-pyruvic transaminase (SGPT, 414 IU/ml), and alkaline phosphatase (ALP, 253 IU/ml) was requested for her. At the end of the fourth month, the patient stopped taking the medicine, and after a few days, clinical symptoms such as anorexia and icteric sclera were initiated. SGOT (1239 IU/ml), SGPT (805 IU/ml), ALP (198 IU/ml), total bilirubin (8.42 mg/dl), direct bilirubin (7.15 mg/dl), PT (17.7 s), international normalized ratio (INR, 1.34), and blood cell count (normal) tests were requested for her. The patient was diagnosed with drug hepatitis and hospitalized for further treatment.

During the first week of hospitalization, liver dysfunction continued, and the INR test results showed progression toward acute liver failure and the emergent need for liver transplantation.

In the ultrasound examination, the liver was normal size, had reduced echo, and showed increased periportal echogenicity indicative of periportal cuffing and increased thickness of gallbladder walls. In addition, a portal vein with a diameter of 8 mm and a peak flow velocity of hepatorenal flow (15 cm/S) were reported.

Abdominal and pelvic computerized tomography (CT) scans with and without oral and intravenous contrast were performed in which the liver had a uniform and normal size and parenchyma; also, no sign of space-occupying mass was seen. The bile duct had a normal appearance and an increase in the thickness of the gallbladder wall was observed; echocardiography was also unremarkable. The extrahepatic bile duct and bile duct of the hepatic lumen were reported normal without evidence of stenosis, dilatation, or filling defect in the magnetic resonance cholangiopancreatography (MRCP). The gallbladder was contracted and mild periportal edema and edema around the gallbladder were observed. A histopathologic survey of the liver biopsy showed cholestasis with moderate-to-severe inflammation and degenerative changes. Binucleated and giant hepatocytes and prominent cholestasis, along with moderate-to-severe mixed inflammatory cell infiltration, including eosinophil, neutrophil, and lymphocytes were seen. Irregular and bulged endothelial cells and apoptosis in hepatocytes with prominent kupffer cells were also noted. In addition, mild fibrosis was identified. Acute viral hepatitis markers were examined, all of which were negative for hepatitis B virus (HBV), hepatitis C virus (HCV), and hepatitis A virus (HAV). Serum copper and ceruloplasmin were normal. Autoimmune hepatitis markers were negative. The patient’s albumin was reported to be 2.1 and the patient’s vitamin D level was low. The patient underwent relative rest and supportive treatments. The patient’s appetite was still disturbed, but there was no evidence of bleeding in the skin, mucous membranes, and other places. As presented in Table 1, the patient’s liver tests changed from the tenth day, the liver transaminase enzymes gradually started to decrease, and the INR test also started to decline and reached below 1.5. After 2 weeks of hospitalization, she was discharged with partial recovery and a follow-up visit was scheduled for a week later. After follow-up visit, the patient’s appetite had improved and she had no particular problems. Liver transaminase enzymes had decreased to less than 200, and the patient’s INR was 1.4. One month after discharge, she was subjected to laboratory tests again, which showed an improvement in liver function, and complementary recommendations were given to her.

**Table 1:** The results of different lab tests by day

Variable	First day of hospitalization	Third day of hospitalization	Fifth day of hospitalization	Tenth day of hospitalization	Twelfth day of hospitalization	Fourteenth day of hospitalization	1 week after discharge	1 month after discharge	Reference range
WBC	5.6	5.6	6	6.5	5.7	5.4	6.5	7.4	4.5–11 (× 10^3^ per μl)
Hb	12.4	11.3	12.3	12.6	11.9	12.1	13	13.1	14–16.5 gm/dl
PLT	361	274	333	298	315	353	280	350	440–150 (× 10^3^ per μl)
Bilirubin (total)	16.4	14.6	15.6	14.7	8.7	6.2	4	2	0.3–1 mg/d
Bilirubin (direct)	6.3	5.5	5.7	5.3	4	3.4	1.6	0.8	0.1–0.3 mg/dl
BUN	10	10	10	14	16	16	21	25	6–21 mg/dl
Cr	0.8	0.8	0.8	0.74	0.9	0.8	0.74	0.7	0.7–1.4 mg/dl
SGOT	1179	730	630	491	452	237	190	110	7–55 μ/l
SGPT	960	513	451	293	258	211	193	102	8–48 μ/l
ALP	398	307	301	304	254	245	283	282	40–129 μ/l
PT	14.9	19.1	21.3	16.4	15	15	14	15	11–13.5 seconds
INR	1.43	2.14	2.56	1.67	1.44	1.28	1.4	1	0.8–1.1
Albumin	2.3	–	–	–	–	–	3.1	–	3.5–5 g/dl

*WBC* white blood cells, *Hb* hemoglobin, *PLT* platelet count, *BUN* blood urea nitrogen, *Cr* creatinine, *SGOT* serum glutamic oxaloacetic transaminase, *SGPT* serum glutamic-pyruvic transaminase, *ALP* alkaline phosphatase, *PT* prothrombin time test, *INR* international normalized ratio

## Discussion and conclusions

Various factors can cause damage to the liver, such as infectious agents, toxins, autoimmunity, and drugs. Drug-induced liver injury (DILI) is the leading cause of acute liver. Age, gender, and pregnancy, as well as comorbidities such as obesity, diabetes, and underlying liver disease are outstanding risk factors for developing DILI [1, 3]. Tofacitinib interferes with the JAK-STAT signaling pathway and suppresses the production of inflammatory mediators by inhibiting Janus kinase 1 (JAK1) and Janus kinase 3 (JAK3) enzymes. In 2012, this drug was approved by FDA to treat several complications such as rheumatoid arthritis, psoriatic arthritis, and ulcerative colitis. According to research, tofacitinib is effective in treating of alopecia areata [4]. However, this drug can affect liver enzymes, and due to its side effects, the use of more than 5 mg twice a day is strictly prohibited [5–7]. According to clinical trial studies, serum aminotransferase elevations occurs in 28–34% of patients who received tofacitinib. In these studies mild and transient liver involvement was described, and no case of severe liver damage was reported. Moreover, our patient experienced liver failure following prescription of tofacitinib. If emergency interventions were not performed immediately, it could have had severe consequences for the patient. The exact mechanism of liver injury is not elucidated. It is suspected that liver damage may be associated with producing of toxic or immunogenic intermediates [8]. In addition, recent studies emphasize that tofacitinib can increase the risk of infection and cardiovascular disorders. The use of tofacitinib and other JAK/STAT inhibitors in diabetic patients and in those who have a history of cardiovascular disorder and stroke is advised with caution [9–12].

This report showed that long-term use of this drug should be avoided due to its hepatotoxicity, despite its therapeutic effects in several disorders. To our knowledge, this is the first report regarding clinical manifestations of tofacitinib-associated DILI in which the severity and outcome of liver injury were characterized in detail. It could be a valuable reference for differential diagnosis, early detection, timely management, and effective treatment for tofacitinib-induced hepatotoxicity.

## Data Availability

Not applicable.
